# A *narrow* gene encoding an extracellular matrix is involved in the formation of the footpad hairs in *Drosophila melanogaster*

**DOI:** 10.17912/micropub.biology.000804

**Published:** 2023-04-07

**Authors:** Kohei Tanaka, Ryunosuke Minami, Ken-ichi Kimura

**Affiliations:** 1 Laboratory of Biology, Sapporo Campus, Hokkaido University of Education, Sapporo 002-8502 Japan; 2 Present address: Department of Advanced Medical Science, Asahikawa Medical University, Asahikawa 078-8510 Japan

## Abstract

*Drosophila melanogaster* is an insect that can walk on smooth surfaces, and its tarsal segments bear a pair of footpads that are equipped with spatulate-shaped hairs (setae). We found that *narrow ^B^* ( *nw ^B^* ) mutants, an allele of the *nw* gene, were unable to climb smooth surfaces, due to the destruction of the footpad hair tips. The mutant hair tips were damaged during molting from the pupal cuticle at eclosion. Thus, the *nw* gene encoding a secretory protein that serves as an extracellular matrix is implicated in the formation of the footpad hairs.

**Figure 1. Behavioral and morphological characterization of the narrow mutant:**
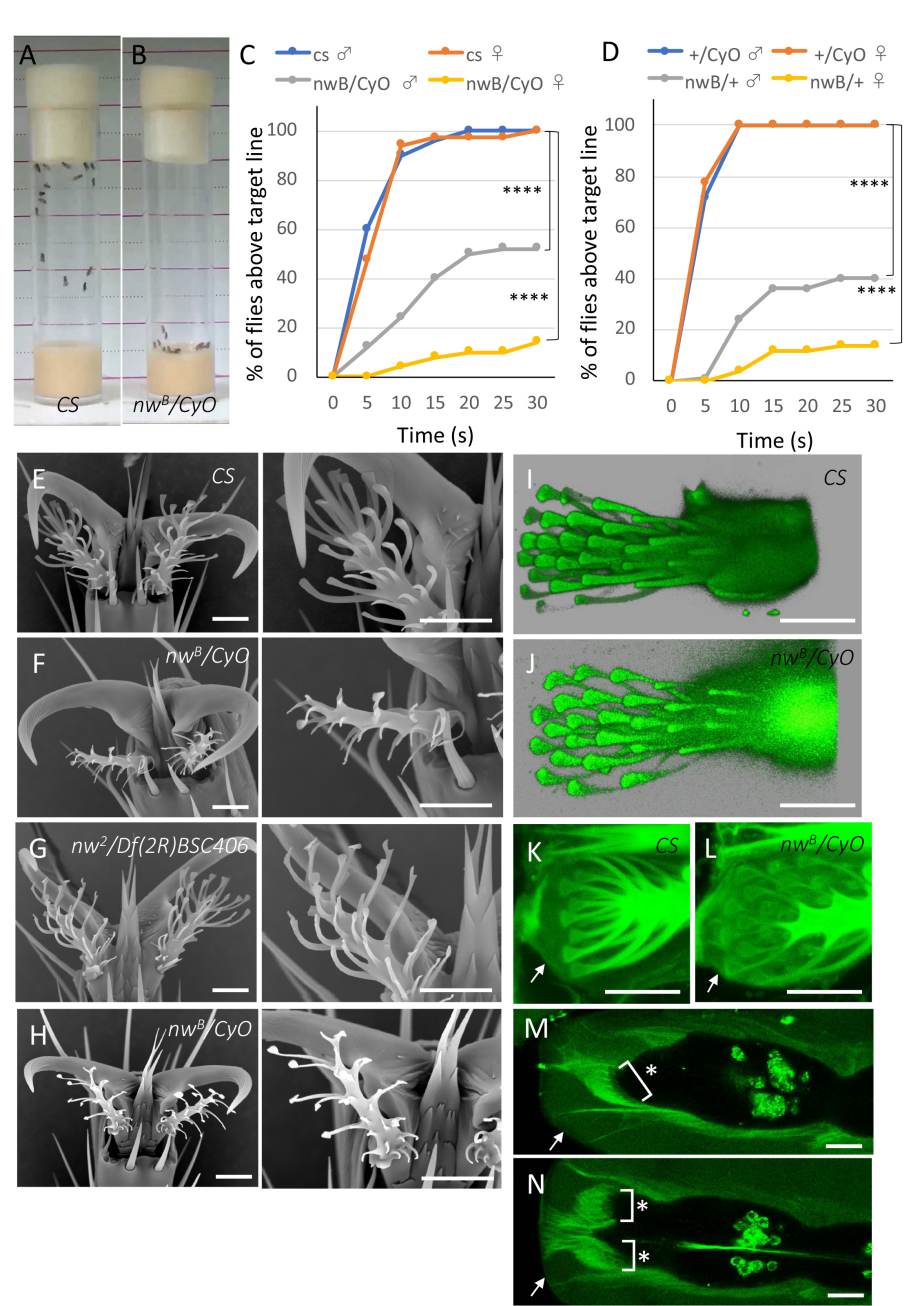
The climbing behavior of wild-type flies ( *CS* strain) and mutant flies ( *nw ^B^ /CyO* strain) was evaluated in fresh culture bottles in (A) and (B), respectively. Climbing assays in *CS* and *nw ^B^ /CyO* were conducted and the cumulative distribution curves of the fraction of flies above the target line were described in (C). The Kolmogorov-Smirnov test was employed to compare the distributions of *CS* and *nw ^B^ /CyO* males and their corresponding females, with a significant difference (****p<0.0001). Additionally, the cumulative distribution curves of the fraction of flies above the target line were described in *CyO/+* and *nw ^B^ /+* and the Kolmogorov-Smirnov test was utilized to compare the distributions of *CyO/+* and *nw ^B^ /+* males and their corresponding females, with **** p<0.0001 (D). Scanning electron microscopy (SEMs) images of footpad hairs were presented in wild-type flies ( *CS* strain) (E), in *nw ^B^ /CyO* (F), in *nw ^2^ /Df(2R)BSC406* (G) and in *nw ^B^ /CyO* just after eclosion (H). The magnified versions of the corresponding left panels were displayed in the right panels. Three-dimensional (3D) images of footpad hair cells at 44 hr APF revealed no significant differences between wild-type (I) and *nw ^B^* mutant flies (J). Footpad hairs covered with pupal cuticle (arrows) just before eclosion were not defective in either wild-type (K) or *nw ^B^* mutant flies (L). At 30 hr APF, the GFP-labeled Narrow protein was observed to be localized apically at the tip of the tarsus, covering the footpad hair cells, as well as forming fibrous connections between the tips of the tarsus and pupal case (arrows) (M, N). (M) illustrated a side view and (N) depicted a horizontal view. The black area outlines the epidermal cell layer of the tarsus and * indicates the region of the footpad cells. The left side of the images shows the distal tips of the tarsus. All images in (E-N) were accompanied by a bar scale of 10μm.

## Description

Insects that are able to climb on smooth surfaces possess numerous fine hair-like cuticular structures on their legs, known as footpads, functioning as adhesion organs (Gorb, 2001). These footpads are enabled to adhere to smooth surfaces due to Van der Waals, Coulomb, capillarity and viscosity forces mediated by a liquid substance secreted from the body (Langer *et al.* 2004, Hosoda *et al.* 2022). Thus, the footpad is known to play an important role in adhesion to smooth surfaces, yet its mechanism of formation remains poorly understood (Kimura *et al.* , 2020, Kimura and Hosoda, 2021). *Drosophila melanogaster* , an insect capable of walking on smooth surfaces, possesses tarsal segments that bear a pair of footpads equipped with spatulate-shaped hairs (Ferris,1950. Hüsken *et al.* , 2015, Kimura *et al.* , 2020). This study aimed to elucidate the gene involved in footpad formation in *Drosophila* .

We found a *narrow* ( *nw* ) mutant strain of *nw ^B^ /CyO* that could not climb smooth surfaces. Contrary to the wild-type strain flies that were able to climb the wall of the culture bottle (Fig.1A), the flies of *nw ^B^ /CyO* meandered erratically at the bottom of the culture bottle with little to no individuals reaching the top (Fig.1B). To evaluate the climbing ability on smooth surfaces, a climbing assay was employed. In the wild-type flies, both males and females were able to climb up within 10 sec (Fig.1C, Movies 1 and 2), while, in the *nw ^B^ /CyO* line, the females were hardly able to climb up, and around 50% of the males were partially successful (Fig.1C, Movies 3 and 4). To ascertain whether this defect was attributed to the *nw ^B^* mutation or the *CyO* one, the *nw ^B^ /CyO* line was crossed with the *CS* line and the siblings were subsequently subjected to the climbing assay. The offspring of the *CyO* /+ genotype had the same climbing prowess as the *CS* flies, whereas the *nw ^B^* /+ offspring were unable to ascend as the *nw ^B^ /CyO* flies (Fig.1D). Thus, it was concluded that the climbing defect was caused by the *nw ^B^* mutation.

The reduction in the climbing ability of the mutant may be attributed to abnormalities in negative geotaxis, motor ability, and the structure of the footpad. Of these, if the decrease is due to an abnormality of the footpad, it should be possible to climb on rough surfaces without relying on the footpad, utilizing only the claws. To confirm this hypothesis, the filter paper was placed in a glass culture bottle and the climbing of the mutants on the rough surface was examined. It can be seen that the mutants climbed similarly to the wild-type, albeit at a slightly slower speed (Movies 5 and 6). Consequently, we concluded that the decrease in the climbing ability of the mutant is caused by the abnormality of the footpad, not the abnormality of negative geotaxis or motor ability.

Subsequently, we examined the external morphology of the footpad. In the wild-type, each tip of the hair exhibited a spatulate form (Fig.1E). Conversely, in the mutant footpads, certain hairs displayed shortened lengths and lacked the expected spatulate structure (Fig.1F). Comparable defects in footpad hairs were observed in another allele of *nw* , *nw ^2^* mutation. The mutants of *nw ^2^* over *Df(2R)BSC406* displayed a mild phenotype with some typical hairs, yet the tips of the spatulate structures were often fractured (Fig.1G).

In order to investigate when and how the footpad abnormalies manifest in the *nw ^B^* mutant, we compared the footpad formation process in the wild-type with that of the mutant. The adult leg is formed during the pupal stage, which typically lasts around four days in *Drosophila* . To gain a better understanding of the process of footpad formation, we employed a strain in which the cell membranes of the footpad-forming cells were labeled with GFP. At 40 hr APF (after puparium formation), spatulate structures at the tips of the processes of the hair cells were observed, exhibiting a morphology similar to that of adult hairs, in both the wild-type (Fig.1I) and the mutant hair cells (Fig.1J). Consequently, the *nw ^B^* mutant exhibited no defects in the formation processes of cellular protrusions.

On the second day of pupal development, the footpad's shape is formed by the hair cells and subsequently enveloped by a hard cuticle (Kimura *et al.* , 2000, Kimura and Hosoda, 2021). Then, the cuticular structure of the hair was monitored in pharate adult specimens, through the autofluorescence of the cuticle. Both the wild-type and the *nw ^B^* mutant displayed normal length and spatula structure (Fig.1K and L, respectively). Then, a scanning electron microscope was employed to analyze the morphology of the adult footpad within 10 minutes of eclosion. The wild-type exhibited a normal footpad, whereas the *nw ^B^* mutant displayed shortened hairs with broken tips (Fig.1H). Therefore, it can be concluded that the shape of the *nw ^B^* mutant's footpad is formed normally until eclosion, however, abnormalities become apparent immediately after eclosion, likely due to the destruction of the hair tips during molting from the pupal cuticle.

The *nw* gene encodes a secreted protein with a C-type lectin domain that functions as an apical extracellular matrix (ECM) component (Ray *et al.* , 2015). The expression of the GFP-labeled Narrow protein (Ray et al., 2015) driven by the *Dll-Gal4* driver was examined to understand the distribution of the Nw protein during footpad formation ( Fig. 1L, M ). At 30 hr APF，the GFP-labeled Narrow protein was localized apically at the tip of the tarsus and was found to cover the footpad hair cells. Moreover, fibrous linkages between the tips of the tarsus and pupal case were also detected.

In this study, the *nw ^B^* mutant displayed normal footpads until just before eclosion, however short hairs were already evident immediately after eclosion. This postulates that the footpad hairs are damaged during eclosion, possibly due to the faulty Narrow protein that results in a more fragile cuticle of the footpad than typical. In the future, further investigation into the expression patterns of the *nw* gene and its product, in addition to an analysis of the aberrant cuticle, should enable us to gain insight into the function of the ECM, Narrow, in the formation of the footpad hairs.

## Methods

**Fly strains**

Flies were raised on cornmeal-yeast medium at 25°C under constant illumination. *Canton-S* ( *CS* ) flies were employed as the wild-type strain. A line of a *nw* mutation, *nw ^B^ /CyO, P{Gal4-Kr,C}DC4, P{UAS-GFP,S65T}DC8* (herein designated as *nw ^B^ /CyO* ) was utilized. Another allele of *nw ^2^ /CyO* was crossed with *Df(2R)BSC406/SM6a* , and the ensuing F1, *nw ^2^ / Df(2R)BSC406* , was assessed. To follow the progress of footpad formation during metamorphosis, footpad hair cells were labeled with membrane-localized GFP. A strain of *UAS-mCD8::GFP; svp ^NP0724^ -Gal4/TM6b* and *nw ^B^* */UAS-mCD8::GFP; svp ^NP0724^ -Gal4/TM6b* were used as the wild-type and mutant, respectively. The distribution of the Nw protein during footpad formation was examined in *w; Dll ^em212^ -Gal4/CyO ; UAS-NwSc-GFP/TM6b* flies.

**Climbing assay**

To evaluate the climbing ability on smooth surfaces, adult males and females were collected within 20 hours of eclosion and maintained in vials containing Kimwipe paper moistened with 20 mM sucrose solution. Ten individuals, aged 24-44 hours of eclosion, were transferred to a glass culture bottle, gently tapped to the bottom of the tube, and video recorded (Evrio JVC, Japan) for one minute to assess their climbing ability. This procedure was repeated five times on each gender and different genotypes (in total, 50 individuals). The proportion of flies that had crossed the 8 cm line every five seconds was represented over the course of the assay. To assess their climbing ability on rough surfaces, flies were placed in a glass culture bottle containing a piece of filter paper and examined for their ability to climb on the paper. The flies used were of genotypes *+: +* ( *CS* strain), as a control, and *nw ^B^ /CyO* for the experimental strains.

**Observation of footpad using scanning electron microscopy**

The footpad structure of *D. melanogaster* was studied using a scanning electron microscope (SEM). The fly legs were initially fixed with 3.7 % formaldehyde, followed by a comprehensive wash in distilled water and a serial dehydration process with ethanol. Tert-butyl alcohol was then used to replace the ethanol before the samples were cooled to 4°C and subjected to freeze-drying (VFD-21S, Vacuum Device Inc.). Subsequently, an ion coater (Neo coater MP-19010 NCTR, JEOL) gold-coating was applied, and the specimens were observed using SEM (TM3000 Miniscope, Hitachi).

**Observation of developing footpad during metamorphosis using confocal microscopy**

Images of the developing footpad during the pupal stage were acquired from *UAS-mCD8::GFP; svp ^NP0724^ -Gal4/TM6b* and *nw ^B^ /UAS-mCD8::GFP; svp ^NP0724^ -Gal4/TM6b* flies at 44 hr APF, and *w; Dll ^em212^ -Gal4/CyO ; UAS-NwSc-GFP/TM6b* flies at 30 hr APF. Following the removal of puparium, the pupae were secured to a glass coverslip and imaged using a Leica TCS SPE confocal microscope. Images of the footpad hair structure at the pharate stages prior to eclosion were obtained by cuticle autofluorescence using a confocal microscope.

**Statistical analysis**

The Kolmogorov-Smirnov test was applied to evaluate the statistical significance of the difference between the cumulative distribution curves of the fraction of flies versus time. A p value of less than 0.01 was considered to be statistically significant.

## Reagents

**Table:** 

Fly Strains		
Name	Genotype	Stock ID
* nw ^B^*	* nw ^B^ /SM5 *	DGGR 105809
* nw ^2^*	* In(2L)Cy In(2R)NS, DuoxCy / nw ^2^*	DGGR 101514
*Df(2R)BSC406*	* w ^1118^ ; Df(2R)BSC406/SM6a *	BDSC 24430
* svp ^NP0724^ -Gal4 *	w*; P{GawB}svp ^NP0724^ / TM3, Ser ^1^	DGGR 103727
*UAS-mCD8::GFP*	y ^1^ w*; P{w[+mC]=UAS-mCD8::GFP.L}LL5	DGGR 108068
* Dll ^em212^ -Gal4 *	* w ; Dll-GAL4 ^em212^ / CyO ; UAS-GFP /TM6b *	Gorfinkiel et al., 1997
*UAS-NwSc-GFP*	* w ^1118^ ;; UAS=Nw-Sc-GFP-1/TM3, Sb *	Ray et al., 2015
BDSC, Bloomington Drosophila Stock Center; DGGR, KYOTO Drosophila Stock Center		

## Extended Data

Description: Movie 1 Climbing behavior on smooth surface in wild-type male flies Wild-type female flies (CS strain) are able to climb the smooth wall of the culture bottle.. Resource Type: Audiovisual. DOI: 10.22002/y5hpy-2yc37

Description: Movie 2 Climbing behavior on smooth surface in wild-type female flies Wild-type male flies (CS strain) are able to climb the smooth wall of the culture bottle.. Resource Type: Audiovisual. DOI: 10.22002/g37cw-3bn62

Description: Movie 3 Climbing behavior on smooth surface in nwB /CyO male mutants About half of the nwB /CyO male mutants are unable to climb the smooth wall of the culture bottle.. Resource Type: Audiovisual. DOI: 10.22002/yg1hz-5by86

Description: Movie 4 Climbing behavior on smooth surface in nwB /CyO female mutants More than half of the nwB /CyO female mutants are unable to climb the smooth wall of the culture bottle.. Resource Type: Audiovisual. DOI: 10.22002/zgyfy-zde90

Description: Movie 5 Climbing behavior on rough surface in wild-type female flies Wild-type female flies of the CS strain are able to climb the rough wall of the filter paper.. Resource Type: Audiovisual. DOI: 10.22002/jhkfp-xxs50

Description: Movie 6 Climbing behavior on rough surface in nwB /CyO female mutants nwB /CyO female flies are able to climb the rough wall of the filter paper.. Resource Type: Audiovisual. DOI: 10.22002/57g85-vnh44
